# Translational research of temporomandibular joint pathology: a preliminary biomarker and fMRI study

**DOI:** 10.1186/s12967-019-02202-0

**Published:** 2020-01-13

**Authors:** Andre Barkhordarian, Gary Demerjian, Francesco Chiappelli

**Affiliations:** University of California Los Angeles, School of Dentistry, Division of Oral Biology and Medicine, Los Angeles, USA

**Keywords:** Temporomandibular joint (TMJ), Temporomandibular joint disorders (TMD), Functional magnetic resonance imaging (fMRI), Trigeminal ganglion (TG), Microglia cells, Blood oxygenation level dependent (BOLD), Dental orthotic, Default mode network (DMN), Fractional anisotropy (FA), Arterial spin labeling (ASL)

## Abstract

**Background:**

The temporomandibular joint (TMJ) is well innervated by braches of the trigeminal nerve. The temporomandibular joint disorders (TMD) can cause neural-inflammation in the peripheral nervous system (PNS) at the site of injury, or compression, and may have systemic effects on the central nervous system (CNS). Neural-inflammation causes elevations in cytokine expression and microglia activation. When the site of injury, or compression is treated, or relieved, neural inflammation is reduced. These changes can be seen and measured with fMRI brain activities.

**Methods:**

For this study, patients with comorbid TMD and systemic/neurologic conditions were compared using clinical diagnostic markers, inflammatory, pain, tissue destruction enzymatic biomarkers, and functional magnetic resonance imaging (fMRI) activity of the brain, with and without a custom-made dental orthotic.

**Results:**

Our results showed a correlation between the clinical diagnosis of the pathological TMJ, biomarkers and the fMRI study. There was a marked elevation of biomarkers in samples taken from TMJ of patients who were clinically diagnosed with TMD. The fMRI study of TMD patients showed an abnormal hyper-connected salience network and a diminished blood flow to the anterior frontal lobes when they did not wear their customized dental orthotics.

**Conclusions:**

Our findings highlight the importance of TMJ-CNS connections and use of fMRI as an investigative tool for understanding TMD and its related neurological pathologies.

## Background

The temporomandibular joint (TMJ) is a complex joint, which comprises the relationship of the mandibular condyle, articular disc and glenoid fossa of the temporal bone. The articular disc divides the TMJ into compartments with distinct mechanical functions. During the opening motion of the mouth, the mechanism of action between the articular disc is to traverse in the anterior direction across the surface of the glenoid fossa, while the mandibular condyle is rotating under the concave surface of the disc [1]. The TMJ is innervated by the masseteric nerve anteriomedially and deep temporal nerve anteriolaterally. The auriculotemporal (AT) nerve supplies the TMJ from the posterior side and branches out to the medial and lateral aspects of the joint, while the masseteric and posterior deep temporal nerves supply the anterior aspect of the joint [2]. Pathologies affecting the TMJ are termed as temporomandibular joint disorders (TMD), a common condition involved in orofacial pain. Early investigators indicated 1–75% of the populations had at least one objective sign of TMD, while 5–33% reported subjective symptoms with a higher prevalence of women ages 20–40 [3]. TMD exists in conjunction with many comorbid conditions such as headaches, tinnitus and hearing loss, but can also include systemic disorders such as irritable bowel syndrome, ulcers, high blood pressure, allergies, cardiovascular disease, fibromyalgia, chronic fatigue syndrome, arthritis, neck and back pain [4–7]. Recent studies show that neurological disorders, such as Parkinson’s Disease, Cervical Dystonia, Tourette’s syndrome may have a root cause in TMJ related disorders. Undiagnosed TMJ disc dislocation such as distal condylar displacement and associated compression/irritation of the AT nerve may be due to bone loss, trauma, bruxism and other pathological etiologies [8, 9]. Therefore, any neural interaction of the AT nerve can lead to a broad array of disorders such as neurologic, dystonic and neuromuscular disorders. TMD and its comorbid conditions are the correlation of the TMJ neural integration within the brainstem centers via the sensorimotor system. The neural networks are intertwined controlling balance and coordination. TMD can be one of many physical manifestation of a more extensive set of systemic problems [10].

This study is positioned at the cutting-edge domain of bio-clinical research, and its focus and intent is patient-centered translational research and effectiveness where the patient’s health and well-being is at the center of importance. Literature and clinical studies have shown that subconscious stress related behaviors such as bruxism (clenching and grinding) as well as jaw related trauma could cause derangement of the jaw joints [11]. This will result in bone loss and subsequent irritation and compression of the trigeminal nerve leading to local and systemic inflammation and numerous symptoms and pathologies through the CNS, which could be detected by elevation of certain immune, pain and tissue destruction biomarkers in synovial fluid and saliva. The resulting inflammatory response is controlled in part by a bidirectional communication between the brain and the immune systems. It involves hormonal and neuronal mechanisms by which the brain regulates the function of the immune system. Cytokines on the other hand, allow the immune system to regulate the brain. In healthy individuals, this bidirectional regulatory system forms a negative feedback loop, which keeps the immune system and the CNS in balance. Many studies have shown that neuro-inflammation might spread from the site of nerve entrapment, to the CNS and the brainstem centers where it could act as physiological driver for aberrant reflexive behaviors, as well as changes within the nervous system. Examining the anatomy of the trigeminal nerve, we see connections to the CNS and its sensory branches that innervate most visceral structures such as the lung, the gastrointestinal tract, heart and immune cell rich tissues and organs such as spleen and lymph nodes. Therefore, TMD symptoms can be local and specific, and or varied and systemic such as: comorbid neurological conditions, including cervical dystonia, Parkinson’s disease, Tourette syndrome, Blepharospasm and complex regional pain syndrome (CRPS) [8].

We know now that infection, neurological damage and diseases elicit a local inflammatory response and activate the immune system. In the CNS, this response is driven by the microglia cells (resident immune cells in the brain region). In healthy systems and normal conditions, activation of microglia will promote recovery and the inflammatory response will resolve. Microglia will go back to its resting state monitoring the microenvironment of the brain. However, mounting evidence has shown that sustained systemic inflammation can initiate neurological diseases such as Alzheimer’s disease and Parkinson’s disease. The main cause is believed to be the hypersensitive state which microglia can enter upon continuous stimulation, which will result in excess production of pro-inflammatory mediators upon systemic inflammation, or injury [8]. Inflammation has been implicated in neuronal damage and cognitive impairment [12, 13]. Most primary afferent nociceptive neurons that innervate the head and neck region are located in trigeminal ganglion (TG). Peripheral signals from particular region of TMJ, can be transmitted to CNS directly via glossopharyngeal nerve and vagus nerve resulting in accumulation of neuropeptides. Inflammation of TG results in activation of glial cells, such as microglia, astrocytes and satellite glial cells (SGCs) that are key players in the onset of neurological diseases. In CNS glial cells become activated and stimulate the release of pro-inflammatory cytokines such as TNF-α, IL-1β and IL-6. This results in an increased firing and activation as well as hyperexitability of nociceptive neurons and development of hyperalgesia and allodynia [14–17].

Our interest in the role of pain neuropeptides in clinical manifestation of TMD resulted in the analysis of two principal neuropeptides, the substance P (SP) and the calcitonin gene-related peptide (CGRP), both recognized as biomarkers of pain that are involved in different aspects of pain development and perception. SP is a neuropeptide that is produced in the perikaryon of capsaicin selective primary afferent neurons and is involved in transmission of sensory stimuli to the CNS [18]. Receptor activation by SP results in an increased expression of cyclooxygenase-2 (COX-2), vasodilatation, increased blood flow and permeability, which allows for plasma extravasation and mastrocyte degranulation, releasing histamine and further amplification of processes that activate nociceptors [19]. SP binds to its receptor on lymphocytes, granulocytes and microphages and stimulates them to produce cytokines. Microphages will produce pro-inflammatory cytokines IL-1β, TNF-α and IL-6 as well as inflammatory mediators such as prostaglandin E2 (PGE2) and thromboxane that in turn will sustain production of further SP neuropeptides and will keep feeding the cycle [20–22] both in PNS and CNS [23].

CGRP is a 37 amino acid neuropeptide that is synthesized and released from the sensory neurons. Trigeminal nerve fibers that contain CGRP innervate the synovial membrane, articular disc, periostium and joint capsule of the TMJ [24, 25]. Elevated level of CGRP in TMJ synovial fluid is an indication of impaired mobility and pain associated with arthritis [26] and inflammation [27]. Many studies have shown that injection of CGRP into the TMJ capsule of rats will result in increased expression of proteins that initiate, develop and maintain peripheral and central sensitization by promoting local inflammation and pain that will in time result in its transition from peripheral tissues to the CNS [26, 28, 29]. Through a series of ongoing research that started more than two decades ago it has been established in the literature that an inflammatory state exist in the TMJ of patients with TMD. Elevated levels of pro-inflammatory cytokines (IL-1β, TNF-α and IL-6) were found in the synovial fluid (SF) of TMD patients [30] that needed to be confirmed and explored further. Since then there has been developments in our understanding of inflammation process where as new polarization states have been determined for T-cells. In 2005 Harrington et al. and Park et al. established that Th-17 cells were a true distinct linage of T-cells. Eventually, retinoic acid (RA)-related orphan receptor γ thymus (Rorγt) was identified as the master transcription factor defining Th-17 cells as a distinct lineage [31–33]. Th-17 cells secrete IL-17 (mainly IL-17A and also B, C, D and F). The primary function of Th-17-related cytokines is to modulate induction of many immune signaling molecules. The most notable role of IL-17 is its involvement in inducing and mediating pro-inflammatory responses that results in sustained (chronic) inflammation and bone resorption [34]. Expression of IL-17 cytokine has been reported in patients with temporomandibular osteoarthritis (OA) [35]. In this study we wanted to confirm and expand what has been established in the literature by characterizing proinflammatory cytokines (IL-1β, TNF-α and IL-6) as well as IL-17A as inflammatory biomarkers of TMD.

Our study also involved the emerging field of Osteoimmunology, which refers to the regulatory interaction between bone and immune cells and related pathways. The interplay begins when bone provides the microenvironment that is critical for the development of the hematopoietic stem cells from which all cells of the mammalian immune system derive, and they in turn produce various immunoregulatory cytokines that influence the fate of bone cells. These influences are at molecular and cellular levels and ultimately determine (or alter) forms and functions. This study also involved terminal biomarkers of tissue destruction, the Peptidyl Arginine Deimminase type 4 (PAD-4). PAD-4 is the enzyme that converts arginine into citrulline, a modified form of arginine. Citrullination is the post-translational modification of protein-bound arginine to form the non-standard (non-charged) amino acid, citrulline [36]. PAD enzymes are present intracellularly as inactive enzyme. The intracellular calcium concentrations are much lower than the required calcium concentration for PAD-4 enzyme activity. Conversion of arginine to citrulline residues is carried out either in tightly regulated microenvironments where extraordinarily high concentrations of Ca2+ are present, or in extracellular environment, where there is leakage of enzymes from dying cells [37].

Recently, research has shown that some of the pain processing pathways are activated by social and emotional experiences showing that perhaps the brain uses some of the same pathways for processing pain and salience [38–43]. Salience is defined as the rank ordering of the importance and distinctiveness of a physiological/environmental stimulus by the brain. Pain is not a purely sensory experience. Emotional pain and its relief without using a pain-relieving medication is evidence that salience is able to produce experiences similar to pain.

Functional magnetic resonance imaging (fMRI) is used to assess brain activity in patients with pain and neurological disorders. Brain imaging studies can depict a baseline neural network considered to be involved in the salience network. Brain structures are associated based on their spatio-temporal characteristics and interconnected structures affecting each other’s activity, reflecting a synchronous modulation in the blood oxygenation level dependent (BOLD) fMRI signal.

fMRI signals are produced through hemodynamic changes in blood oxygenation of cortical structure. These BOLD techniques provide an indirect measure of neural activity. Studies of acute and chronic pain have used these BOLD techniques to examine brain responses to a noxious stimulus, task, or pain perception. A common approach to fMRI pain intervention studies link brain activity (i.e., hemodynamic changes) to the presentation of a provocative stimulus in comparison to a baseline or control stimulus to determine brain regions that are involved in the pain response. Functional connectivity assesses the integration of brain activity across distant brain regions, regardless of their structural connectivity. This method is also called “resting state” connectivity and it can also be assessed during a task. fMRI methods can assess connectivity by measuring correlations in the BOLD time-series of activations in different brain regions, and the mutual information between two different profiles of activation using other methods. There are three main methods to assess functional connectivity using the BOLD signal: seed-based, independent components analysis (ICA), and graph theory. We used ICA, which is a model-free method meaning the researcher does not need to select a point of reference region in advance. Instead, the entire four-dimensional fMRI data can be decomposed into time courses and associated spatial maps, describing the temporal and spatial characteristics of the components making up the data. A number of intrinsic connectivity networks (ICNs)-functional networks-can be reliably detected across individuals. ICN connectivity is disrupted in a wide range of psychiatric, developmental and neurological disorders. The ICN that has received the most attention is the default mode network (DMN), a collection of brain regions that have increased activity in the absence of a task also called the ‘task negative’ network. The DMN is generally thought to include the posterior cingulate cortex/precuneus, medial prefrontal cortex, inferior parietal lobules, lateral temporal cortices, and hippocampus [44–48].

Arterial spin labeling (ASL) perfusion MRI imaging is another technique used in Advanced Imaging studies. The perfusion sequence has several benefits over traditional contrast bolus techniques. First, ASL requires no Gadolinium-based contrast agent. Second, ASL can be quantitative. Majority of perfusion techniques available are qualitative, showing relative changes in cerebral blood volume (CBV), cerebral blood flow (CBF), and mean transit time (MTT). Quantification allows for regional and global assessments of cerebral perfusion [49–51]. Diffusion tensor imaging (DTI) can elicit subvoxel, microstructural information within tissues by measuring both the directionality and magnitude of water self-diffusion. The average apparent diffusion coefficient (ADC) measures mean water mobility. Fractional Anisotropy (FA) is scalar measure of relative diffusion anisotropy. Both techniques have been used as surrogate measures for microstructural integrity within the human brain [52, 53].

This study identifies clinical and diagnostic markers of TMD and evaluates their association to the fMRI markers in TMD patients with comorbid neurological disorders before and after orthotic treatment.

We hypothesize patients with TMD will have elevated levels of inflammatory (IL-1β, TNF-α, IL-6, IL-17A), pain (SP, CGRP), and enzymatic biomarker of tissue destruction (PAD-4) in their synovial and whole saliva fluids, which are associated to their clinical diagnostic markers. Furthermore, we hypothesize that TMD patients with neurological conditions, will show changes in brain activity due to normalization of the patient’s neurologic symptoms upon insertion of a dental orthotic.

The specific aims used in this study were: (1) To measure IL-1β, TNF-α, IL-6, IL-17A, SP, CGRP and PAD-4 biomarkers using ELISA kits correlating clinical evaluations and physical examination of the TMJ [including range of motion, intra-oral examination of the hard and soft tissues, palpation of muscles of the head and neck, joint vibration analysis (JVA), electromyography (EMG), cone beam computerized tomography (CBCT) and visual analog scale (VAS) to assess pain levels]. (2) To assess brain activity changes using fMRI advanced imaging techniques such as: MPRAGE to visualize brain morphology, resting state network for analyzing fluctuations of various brain networks, ASL to visualize cerebral blood flow in brain regions, tractography and FA for assessing the structural and myelination integrity of white matter tracts.

## Methods

This study included eleven patients with temporomandibular joint disorders, three of which had neurological conditions. A UCLA IRB was generated and accepted (IRB approved number 13-000027) to receive and process samples at UCLA.

We established a collaboration with the Community Clinic Association of Los Angeles County (CCALAC). Potential TMD patients were identified based on their symptoms at CCALAC associated (member) clinics. They were referred to the dental practice of Dr. Gary Demerjian, who is part of the evidence-based decision practice-based research network (EBD-PBRN) associated with our lab at UCLA School of Dentistry. Patients signed a consent form at the clinic of Dr. Demerjian allowing clinical evaluation of their TMJ joint, and collection of their left and right TMJ synovial fluid as well as whole saliva samples.

Clinical Examination was performed by Dr. Gary Demerjian whose practice is limited to TMJ and Dental Sleep Medicine. Diagnosis was based on clinical examination including a review of the patient’s medical history, clinical symptoms, palpations of the head and neck, intra-oral exam, joint vibration analysis, electromyography, CBCT with an iCAT imaging system, analyzed with Vision software. iCAT is a high frequency, constant potential, fixed anode X-ray source (120 kVp, 3–8 mA). It incorporates a flat panel, amorphous silicon image detector at 20 cm × 25 cm, with a cesium iodide conversion layer. Scan dimensions are 17 cm × 13 cm. BioPak system was used for surface EMG measurements and JVA of the TMJ. Surface EMG pads were used on the bilateral temporalis, masseters, anterior digastric, and sternocleidomastoid. JVA (Electrovibrography) was used to measure and document the disc displacement during the examination. Treatment included fabrication of patient tailored oral orthotic and periodic adjustments.

Left and right TMJ synovial fluid was obtained as part of the lavage of the joint compartments. A 27 gauge (27G × 1/4 inch − 0.4 × 32 mm) syringe with a hypodermic needle was used to aspirate the TMJ. A 2 ml of 2% LIDOCAINE (20 mg/ml) solution was used in the lavage protocol and the amount of samples obtained per joint was in the range of 250–1000 µl (mixture of 2% LIDOCAINE and SF). Patients were also asked to produce fresh whole saliva samples. All samples were collected in sterile tubes containing sterile buffered solution of chelating agents (1 mM EDTA). They were coded and stored frozen until transported on dry ice to UCLA to be stored at − 80 °C until processed. Samples were batch assayed for IL-1β (Qiagen USA; linear limit of detection 17.9 pg/ml), TNF-α (Qiagen USA; linear limit of detection of 21.4 pg/ml), IL-6 (Qiagen USA; linear limit of detection of 14.0 pg/ml), IL-17A (Qiagen USA; linear limit of detection of 28.4 pg/ml), SP (Cayman Chemicals; linear limit of detection of 8.2 pg/ml), CGRP (Cayman Chemicals; linear limit of detection of less than 5 pg/ml) and PAD-4 (Cayman Chemicals; a range of 1–1000 U/ml and LLOQ 31.3 U/ml) with commercially available ELISA kits.

Clinical observation data were correlated with the concentration of the biomarkers (or parameters) when appropriate using Spearman’s rho correlation coefficient. Patients were ranked based on clinical severity of TMD. Comparisons of left and right TMJ (synovial fluids) were performed by Matched-pair *t* test (or rank-sum when non-parametric). The comparison of left and right synovial fluid and whole saliva within each subject was performed using one-way ANOVA followed by Scheffe Post Hoc Comparison with Bonferroni Correction (or when non-parametric kruskal–Wallis and Wilcoxon). Level of significance was set at α = 0.05 for all statistical analyses.

We also collaborated with Medical Imaging Center of Southern California for fMRI and neuroimaging studies. The fMRI study for patients 9–11 was performed using a Siemens Magnetom Verio 3 Tesla MRI clinical open system scanner (Siemens Healthcare GMbH, Erlangen, Germany) and the following sequences were used: (1) MPRAGE. (2) Resting state network. EPI sequences for this resting state BOLD scan was post processed on a 3D workstation. ICA was performed separating out the various networks. (3) ASL. (4) Tractography and FA. In this study, a quantitative volumetric analysis was not done. Images were visually inspected for gross anatomical abnormalities by experienced observers (neuroradiologists), blind to participant identities without using a quantitative regional analysis and normal database. When we refer to morphology of brain in this study, it is the gross anatomic evaluation of the brain qualitatively as oppose to quantitatively.

Diffusion Weighted Imaging (DWI), can be post-processed to create Diffusion Tensor Imaging (DTI) white matter tractography maps. From the diffusion tensor, diffusion anisotropy measures such as, the fractal anisotropy (FA) can be computed, a scalar value (between zero and one) representing an indirect assessment of the myelination integrity of white matter tracts in the brain. A value closer to zero means diffusion is isotropic, unrestricted (or equally restricted) showing loss of tissue organization, the myelin sheath. The corpus callosum, which connects the two cerebral hemispheres, is the largest white matter fiber bundle in the human brain, mostly traveling in a straight line. This is important because whenever there is a curve in white matter an artificial drop in FA occurs. Corpus callosum has been focus of many studies due to its structural characteristics. Many studies have focused on detecting the morphological changes in white matter integrity that occur in various disorders, particularly in Corpus Callosum, which serves as a sample of brain condition [54, 55]. Five regions of corpus callosum: genu, anterior body, mid body, posterior body and splenium were chosen as region of interest (ROI) in this study and were compared with the average value of the same ROI’s obtained from a normal database. The normal database was meticulously complied from 62 healthy controls ranging in age from 17 to 65, all right handed, at the Medical Imaging Center of Southern California using a Siemens Magnetom Verio 3 T MRI clinical open system scanner (Siemens Healthcare GMbH, Erlangen, Germany) excluding subjects with any brain abnormalities, neurological pathology, drug addiction, hypertension and diabetes detected by MRI scan. In order to minimize variations, the MRI scanner and the techniques were standardized by using the same scanner and post processing techniques creating the normal database, and the individual patient analyses. The post-processing for DWI was done on a Philips DynaSuite Neuro offered by Invivo post-processing workstation, which allows combining anatomical images and results, such as DTI or perfusion color maps, fiber tracks, and fMRI activations in a single 2D and 3D display. Diffusion weighted images were loaded on to Invivo workstation. They were first processed by performing a skull mapping, then the images were fused with MPRAGE in order to create tractography (anatomic map of tracts), then color mapping was done using DynaSuite workstation. The software is able to depict white matter bundles by direction. Left to right by this coding is blue, up and down is red, and anterior to posterior is green. ROI’s are taken by manual visual placement in axial plane at midline. The imaging parameters were as follows: Repetition time (TR) = 8400, eco time (TE) = 104, slice thickness = 3.5 mm, b = 0 and 1000 (images are automatically diversified to 1000).

Arterial Spin Labeling (ASL) generates an image by magnetically labeling water molecules as an endogenous tracer as they travel in blood. Selective radiofrequency (RF) irradiation inverts the magnetization of arterial blood water in the region or plane to which it is applied, usually in the neck for brain perfusion, and a downstream measurement is taken as labeled spins exchange in brain. In most ASL methods, the resulting images are compared to control images where inversion pulse is not applied. The difference reveals the perfusion, indirectly related to the quantification of cerebral blood flow (CBF) in units of milliliters of blood per 100 g of tissue per minute [56]. In this study we used a Siemens protocol offered on the Magnetom plus workstation. The 2D acquisition was processed using syngo image processing software offered by Siemens Healthineers. The heat map technique was used where flow is depicted in color-coding from red to dark blue. Red means more flow dark blue means less flow. We window it such that thalami are viewed, or identified bilaterally as red, visualizing blood flow to the brain regions.

Patients 1–8 had clinical examinations; synovial joint fluid and saliva samples were analyzed for correlation. Patients 9–11, who had TMD and comorbid neurological conditions, were clinically evaluated and participated in fMRI studies. A detailed medical history and diagnostic examination report is provided below.

### Patient 9: reflex sympathetic dystrophy (RSD)/complex regional pain syndrome (CRPS)

#### Subject

A 40-year-old female presented with reflex sympathetic dystrophy and dystonia. Her reported symptoms were facial pain, jaw pain, migraine headaches, eye pain, dizziness, fatigue, muscle twitching, neck pain, ringing in the ears, pain when chewing and visual disturbances (limited field of vision).

Her medical history consists of neuralgia, autoimmune disorder, low blood pressure, muscle spasms, injury to the teeth, mouth, face and neck, kidney problems, muscle aches, hypoglycemia, muscle shaking, prior orthodontic treatment, poor circulation, intestinal disorder, bruising easily, chronic fatigue, depression, insomnia, psychiatric care, painful joints, tired muscles and difficulty concentrating.

She indicated moderate bilateral frontal and parietal headaches and severe bilateral temporal headaches lasting for hours, bilateral jaw pain on opening and chewing, jaw pain on the right while at rest, jaw clicking, teeth clenching, pressure behind the eyes, eye pain, blurred and double vision, photophobia, tinnitus, ear pain, difficulty swallowing, limited opening, neck pain, shoulder pain, thyroid enlargement burning tongue and dry mouth.

#### Clinical examination

Moderate pain was elicited upon palpation of left middle and posterior temporalis, and right anterior digastric. Severe pain was elicited upon palpation of bilateral temporalis, right middle and posterior temporalis, bilateral TMJ capsule, bilateral poster joint, bilateral masseter, bilateral posterior digastric, bilateral sternocleidomastoid, bilateral anterior, middle and posterior scalene, bilateral splenius capitus, bilateral trapezius. Provocation test elicited bilateral joint pain. Clinical examination revealed left TMJ click on opening and closing. Mandibular range of motion was 44 mm, maximum protrusion 10 mm, left and right excursions of 8 mm. Skeletal morphology and posture screening revealed facial asymmetry and a higher left shoulder. Intra-oral examination revealed maxillary and mandibular tori. She presented with a class I molar relationship, 4 mm overbite and 3 mm overjet in centric occlusion. Imaging records consisted of a maxillofacial CBCT and fMRI. Imaging examination revealed bilateral flattening on the anterior surface of the condyles, left condylar pitting/cratering, right bifid condyle, left posterior and superior displaced condyle.

### Patient 10: Cervical dystonia

#### Subject

A 32-year-old female presented with pronounced cervical dystonia. Her symptoms were: neck pain, shoulder pain, back pain, fatigue, migraine headaches, headaches, muscle twitching, dizziness, throat pain, sinus congestion and visual disturbances. She suffers from moderate bilateral temporal, occipital and parietal daily headaches lasting for weeks, upper, middle and lower back pain, limited movement of her neck, shoulder pain and stiffness, pain behind the ears, and tingling in the hands. She has sensitivity to light, sound and touch to her head. She receives Botox injections into the neck and shoulder muscles every 3 months. She reports numerous head and spinal injuries throughout her life.

The patient has been diagnosed with Cervical Dystonia, fibromyalgia, Bell’s palsy, and C2–3 radiculopathy. Past history of surgeries are removal of third molars and gall bladder. The patient further reports a history of low blood sugar, muscle spasms, muscle aches and shaking, sinus problems, chronic fatigue, cold hands and feet, depression, dizziness and difficulty concentrating. List of medications taken are Docusate sodium, Fentanyl Transdermal System, Gabapentin, Oxycodone, Savella, Tizanidine HCL, Norgestimate-Ethinyl Estradiol Tablets, and Lunesta.

#### Clinical examination

Dental examination revealed a left TMJ click on opening. Mandibular range of motion measurements revealed maximum inter-incisal opening of 40 mm, maximum protrusive movement of 8 mm, lateral excursive movements of 7 mm right and 5 mm left. Posture screening revealed facial asymmetry, forward head posture and a higher right shoulder in relation to the left. Intraoral examination of the teeth revealed a molar class I relationship with 4 mm overjet and 2 mm overbite. Wear facets were noted on the dentition and missing third molars. Severe tenderness was elicited upon palpation of bilateral temporalis, masseters, lateral capsule, posterior joint space, sternocleidomastoid, anterior digastric, anterior scalene, middle scalene, posterior scalene, occipital, splenius capitus, trapezius.

Diagnostic tests included electromyography, photography, videography and thermography documentation. EMG’s were placed on her bilateral temporalis, masseter, anterior digastric and Sternocliedomastoid muscles, while the patient was seated in a resting position. Right SCM was contracting while in the resting position and upon delivery of the orthotic, her muscle activity normalized on the EMG’s. Photographs were taken demonstrating her cervical dystonia. Video graphic documentation demonstrated her history and progress of care. Thermographic images showed an increase in surface temperature of the head, neck and upper back. CBCT scan of the maxillofacial examination revealed posterior displacement of both condyles, flattening of the posterior portion of both condyles, and pharyngeal airway impingement. MRI study indicated bilateral articular discs displaced in the closed mouth position, with reduction in the open mouth position.

### Patient 11: Sympathetic dystrophy and chronic pain

#### Subject

36-year-old male presented with joint pain in his hands and feet for over 25 years. He complains of daily back pain, neck pain, shoulder pain, throat pain, fatigue, eye pain, jaw pain, facial pain, numbness of his chin and pain when chewing. His frequent symptoms are ear congestion, ear pain and headaches. His history consists of four rear end collisions. He started having migraine headaches 2 years after a roller-skating accident, where he broke his arm during his middle school years. He reports being in pain, his whole life. He has been diagnosed with sympathetic dystrophy, irritable bowel syndrome, acid reflux and paradoxical sphincter contractions.

#### Clinical examination

Clinical examinations revealed sever pain upon palpation of bilateral temporalis, lateral TMJ capsule, posterior TMJ, masseters, anterior digastric, sternocleidomastoid, trapezius, occipital and splenius capitus. Intra-oral examination revealed maxillary and mandibular tori, buccal exostosis, scalloped lateral borders of the tongue, wear facets on the dentition and gingival recessions. Maximum inter incisal opening of 40 mm, right lateral excursion of 10 mm, left lateral excursion of 12 mm and protrusion of 11 mm. Molar relationship of class I on the right and class II on the left, overbite of 4 mm, overjet of 4 mm and the mandibular midline being to the left by 1 mm in relation to the maxillary midline. CT scans of the maxillofacial examination revealed right condylar flattening on the anterior surface and bilateral posterior and superior displacement of condyles in relation to the temporal fossa.

## Results

### Biomarker study for Patient 1–8

Patient 1: showed a significant difference between synovial left and synovial right sides for the IL-1β biomarker. The concentrations of the proteins were below the detection limit of the ELISA kit (mean conc. Left*− 70.29 pg/ml and mean conc. Right − 71.54 pg/ml) and were meaningless.

Patient 2: showed elevation of IL-6 concentration in right synovial fluid, which was significant (Mean conc. Left 69 pg/ml and Mean conc. Right*159 pg/ml and the p value 0.0305). This patient has active bruxism.

Patient 3: showed no elevation in any of the biomarkers.

Patient 4: showed no significant difference between synovial fluid left and right.

Patient 5: showed a significant difference between concentration of TNF-α biomarker between synovial fluid left and synovial fluid right. The concentrations of the biomarkers were below the detection limit of the ELISA kit (mean conc. Left*− 12.33 pg/ml and mean conc. Right − 26.50 pg/ml) and were meaningless.

Patient 6: showed elevation of IL-6 concentration in the left synovial fluid, which was significant (Mean conc. Left* 70.36 pg/ml and Mean conc. Right 0.00 pg/ml and the p value 0.038). Patient also showed elevation of PAD-4 biomarker on the left side, which was significant (mean conc. Left* 10,680 pg/ml and mean conc. Right 2111.2 pg/ml).

Patient 7: showed a higher absorbance value for SP in the right synovial fluid, which was significant (Mean absorbance Left 0.132 and Mean absorbance Right 0.246), but the result must be interpreted in opposite.

The SP kit function in reverse in regards to absorbance and concentration values of biomarkers. The low concentration standard has the highest absorbance.

Patient 8: showed higher absorbance value for SP in the right synovial fluid, which was significant (Mean absorbance Left 0.144 and Mean absorbance Right 0.296). The SP kit function in an opposite fashion the low concentration standard has the highest absorbance. Therefore, the data can be interpreted as elevation of SP on right.

The SP kit functions in reverse in regards to absorbance and concentration values, the low concentration standard having the highest absorbance.

#### The clinical evaluations for patient 1–8 resulted in the diagnosis of the following

Patient 1: Capsulitis, cephalgia, muscle spasm and bruxism (Based on wear facets on teeth).

Patient 2: Bruxism (Based on wear facets on teeth), patient has active bruxism.

Patient 3: Left TMJ disc displacement with reduction, right TMJ osteoarthrosis, crepitus and bruxism (Based on wear facets on teeth).

Patient 4: Cervicalgia, muscle spasm, myalgia, osteoarthrosis, malocclusion and mandibular discrepancy to cranial base.

Patient 5: Bilateral disc displacement with reduction, myalgia of the masseter and bruxism.

Patient 6: Capsulitis, disc displacement without reduction on the left TMJ, bruxism, limited opening, left TMJ chronic degenerative arthritis and right TMJ acute active degenerative arthritis.

Patient 7: Right TMJ disc displacement with reduction, capsulitis, cervicalgia, bruxism and muscle spasm.

Patient 8: Capsulitis, bilateral disc displacement without reduction on the TMJ, bruxism, limited opening, bilateral TMJ chronic degenerative arthritis.

### Patient 9

#### Diagnosis and treatment of Patient 9

**Fig. 1:**
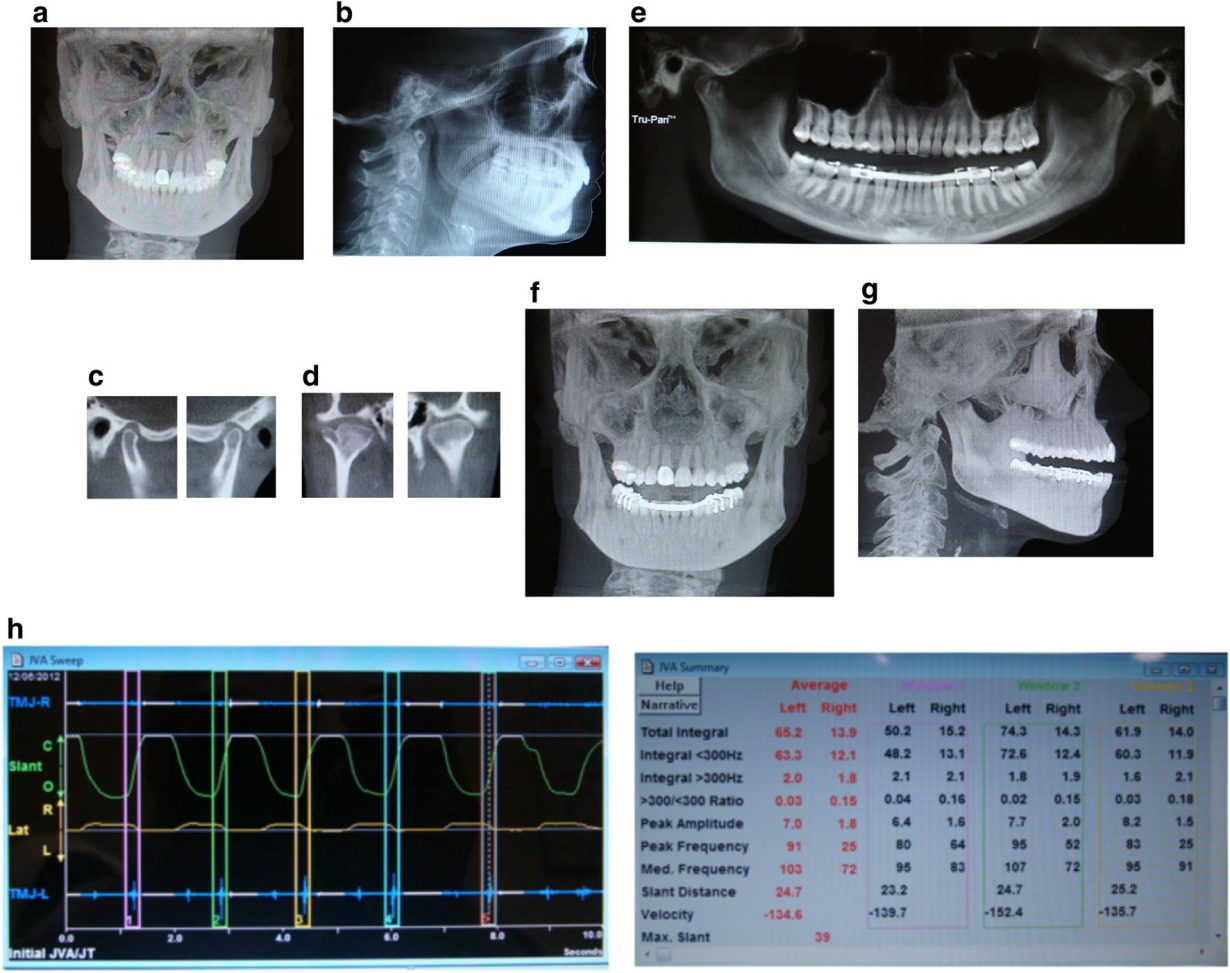
**a** Pre-treatment anterior/posterior cephalometric, **b** pre-treatment lateral cephalometric, **c** sagittal view right and left TMJ, **d** anteroposterior view of right and left TMJ, notice the Bifid shape of the right TMJ, **e** panographic film shows the joint position with the splint in the mouth, **f** frontal view with the splint in the mouth, **g** lateral view with the dental orthotic in the mouth, **h** joint vibration analysis indicating left TMJ disc displacement with reduction

She was diagnosed with TMJ disc displacement with reduction, arthritis, capsulitis, muscle spasm, myalgia and bruxism secondary due to pain. TMJ orthotic was fabricated and adjusted. With the use of her orthotics for 6 months, she reported improvement in her symptoms of over 80% based on visual analogue scale (VAS) (Fig. 1).

#### fMRI study of Patient 9

**Fig. 2:**
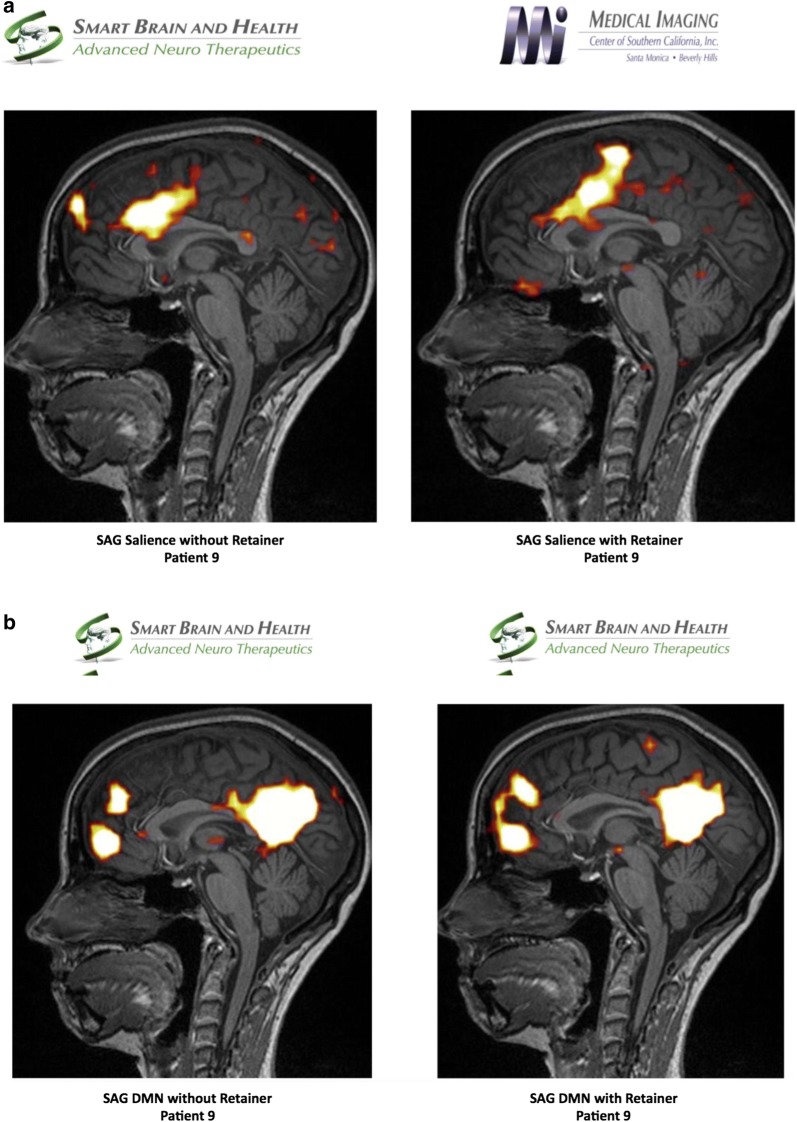

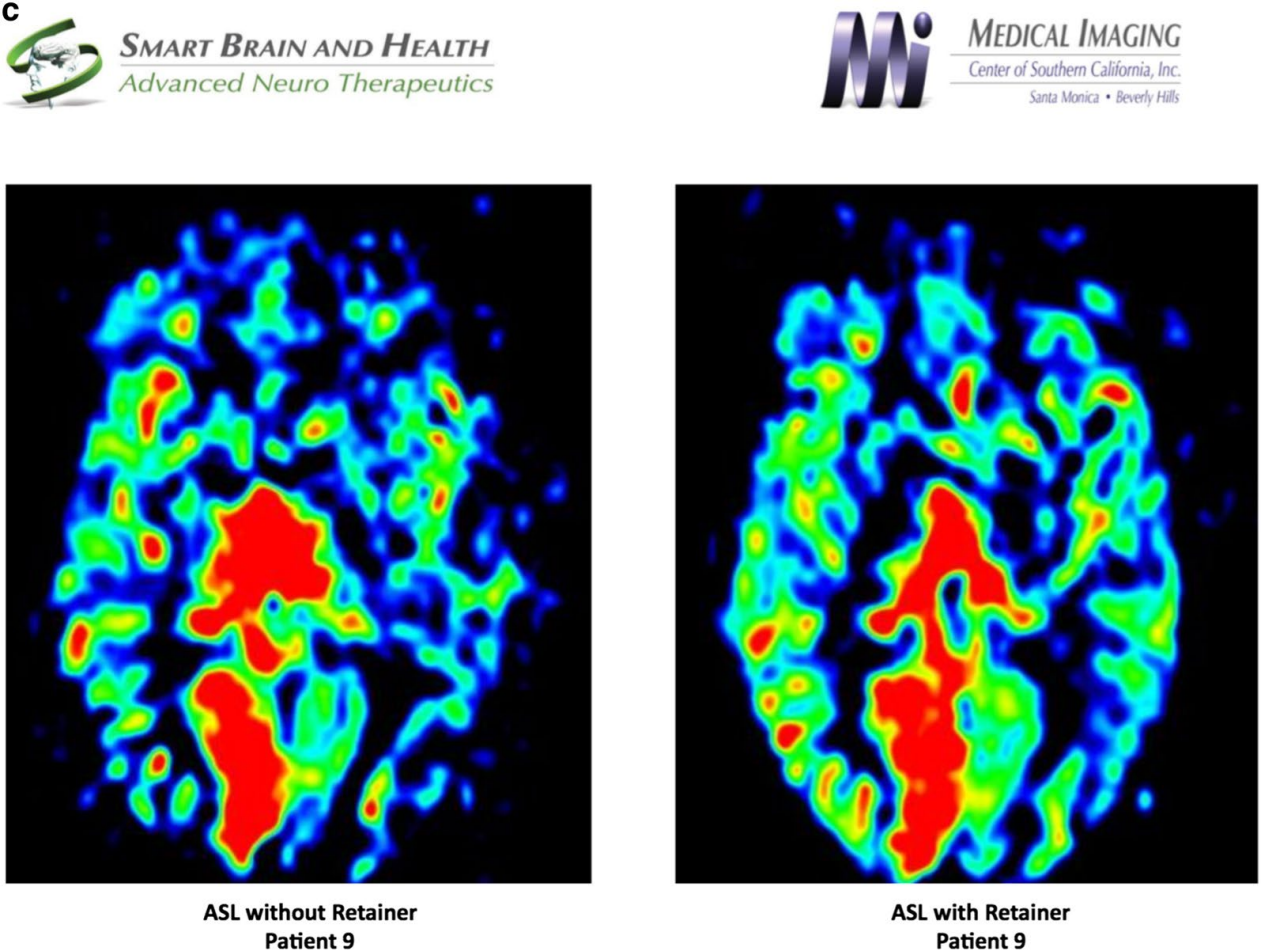
**a** Salient network analysis of patient 9 without and with dental orthotic, **b** default Mode Network analysis of patient 9 without and with dental orthotic, **c** arterial spin labeling analysis of patient 9 without and with dental orthotic

Using independent component analysis, the default mode network was analyzed pre and post positioning of a patient custom-made dental orthotic. The salient network was markedly discrepant between with and without dental orthotic study. Interestingly, the anterior cingulate portion was not significantly different, but there was marked bilateral increased connectivity in the dorsolateral prefrontal cortex in the without dental orthotic study, which incidentally occurred concurrently with severe distress in the patient who complained of pain at the same time. In addition, the medial temporal lobe connectivity was absent in the salient network without the dental orthotic. There was a marked difference in metabolic activity in the anterior frontal lobes of patient’s brain between with and without dental orthotic studies. The blood flow was diminished when patient did not wear the dental orthotic. FA numbers were abnormal in the posterior corpus callosum suggesting a dysfunction in the component of structural connectivity. Incidentally noted was right mastoiditis, which may be secondary to right Eustachian tube dysfunction (Fig. 2).

### Patient 10

#### Diagnosis and treatment of Patient 10

**Fig. 3:**
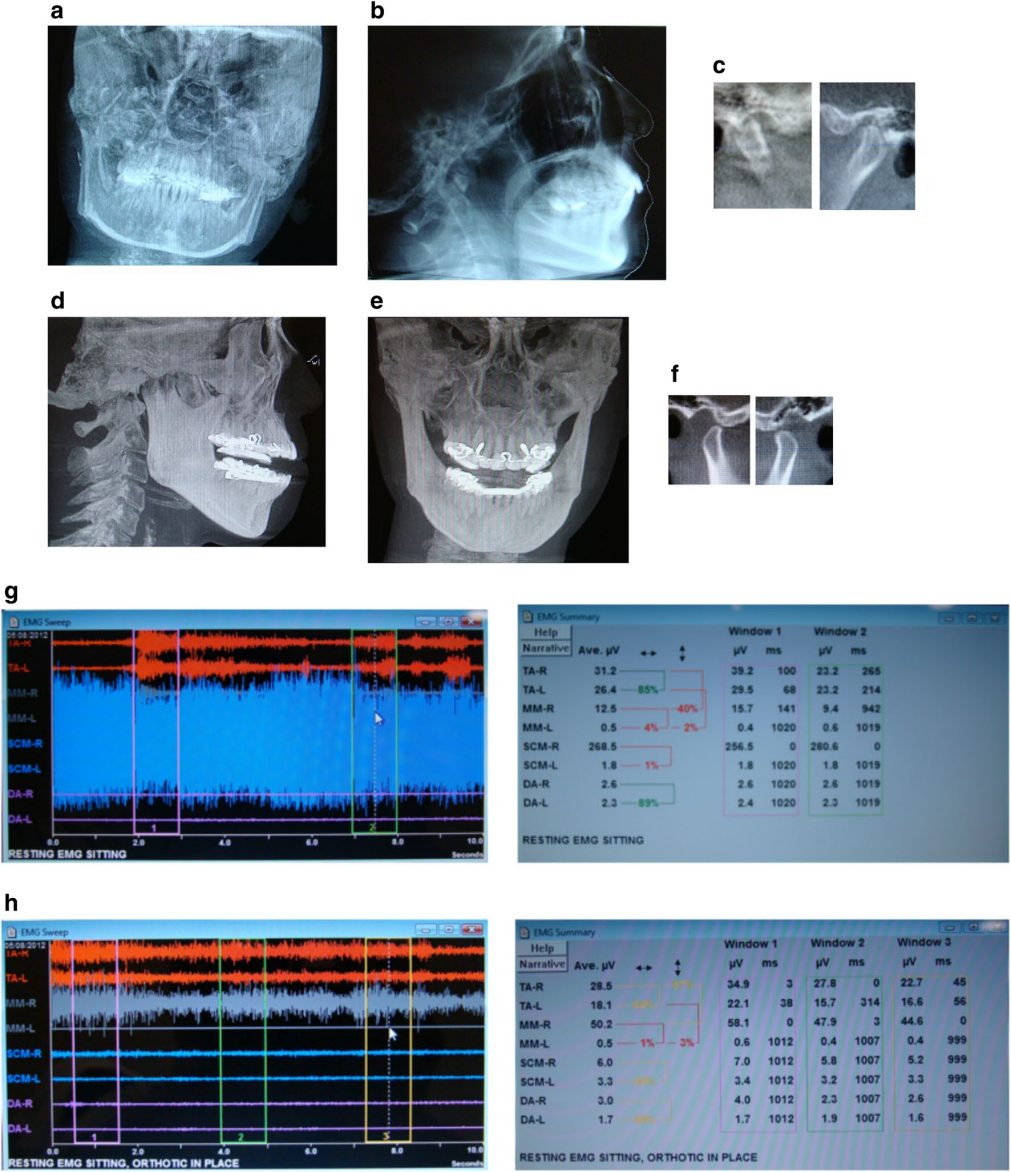
**a** Frontal cephalometric before treatment, demonstrating the head position. **b** lateral cephalometric before treatment, **c** right and Left tomography before treatment, **d** lateral cephalometric demonstrating dental orthotic in the mouth, **e** frontal View, notice the head is more leveled with the dental orthotic, **f** Tomography of the Right and Left TMJ showing condylar position with the dental orthotic in the mouth. **g** EMG pre-treatment, notice the high activity of the right SCM at 286 micro-volts. **h** EMG with splint in, notice the decrease in activity of the SCM muscles. The temporalis and right masseter are slightly elevated and the patient is gently clenching on the dental orthotic

Patient was diagnosed with allodynia of the scalp, hyperalgesia on her neck and shoulders, articular disc disorder, limited mouth opening, muscle spasm, myalgia, cervicalgia, and nocturnal bruxism. During the examination, the patient tested positive to orthopedic changes of the jaw effecting the dystonic pulling. Therefore, an oral orthotic was fabricated to treat the TMJ and stabilize her dystonia. The patient reported resolution of dizziness, visual problems, and migraines on the follow up visits. After 3 months of treatment with the dental orthotic, her symptoms of headaches, neck pain, shoulder pain and Cervical Dystonia greatly diminished (Fig. 3).

#### fMRI study of Patient 10

**Fig. 4:**
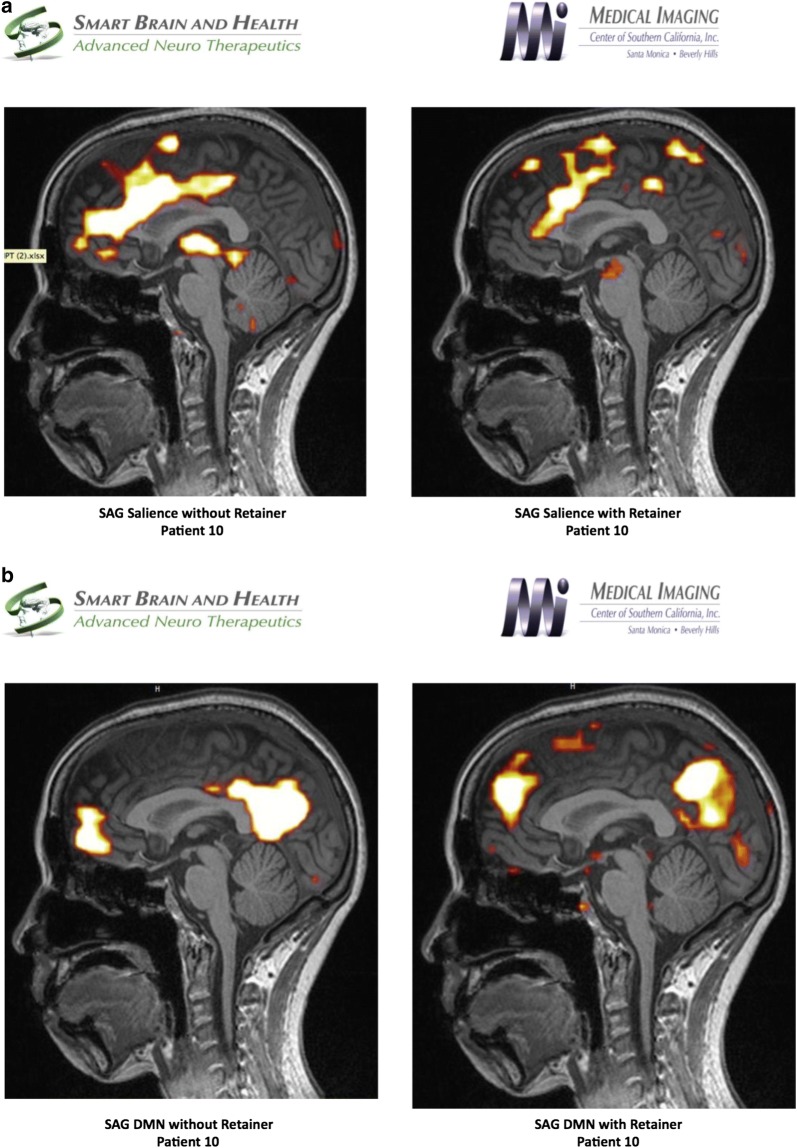

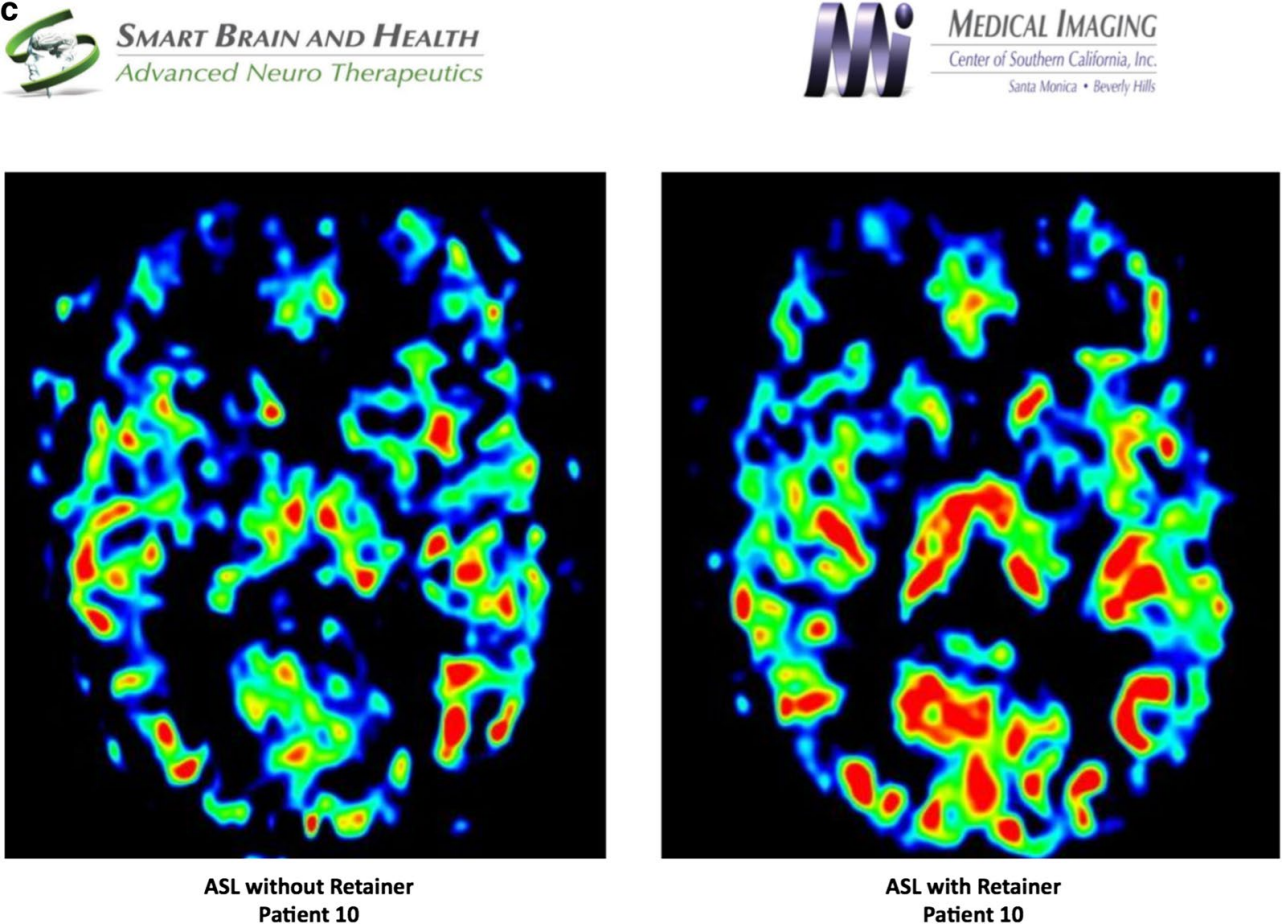
**a** Salient network analysis of patient 10 without and with dental orthotic, **b** default mode network analysis of patient 10 without and with dental orthotic, **c** arterial Spin Labeling analysis of patient 10 without and with dental orthotic

fMRI study was performed using the resting state network, which was further analyzed using independent component analysis to evaluate both the default mode and the salience networks. The study was done with a patient custom-made dental orthotic, which revealed markedly diminished connectivity in the salience network and coincided with the patient feeling much more comfortable without pain. In contrast, when the patient did not wear the dental splint, the fMRI revealed a markedly increased connectivity for the salience network. In addition, the thalami were well visualized as opposed to the study done with the splint. Default mode network revealed a similar pattern of connectivity in the anterior and posterior networks. ASL study revealed diminished flow in the anterior component of the frontotemporal lobe where the dental splint was not used, as opposed to the study when the patient had diminished pain with the dental splint positioned. Diffusion weighted imaging, particularly the FA numbers of the corpus callosum revealed the genu measurement of 0.65, the anterior body 0.33, the posterior body 0.35 and the splenium 0.72. These values are within normal limits for the patient’s age (Fig. 4).

### Patient 11

#### Diagnosis and treatment of Patient 11

**Fig. 5:**
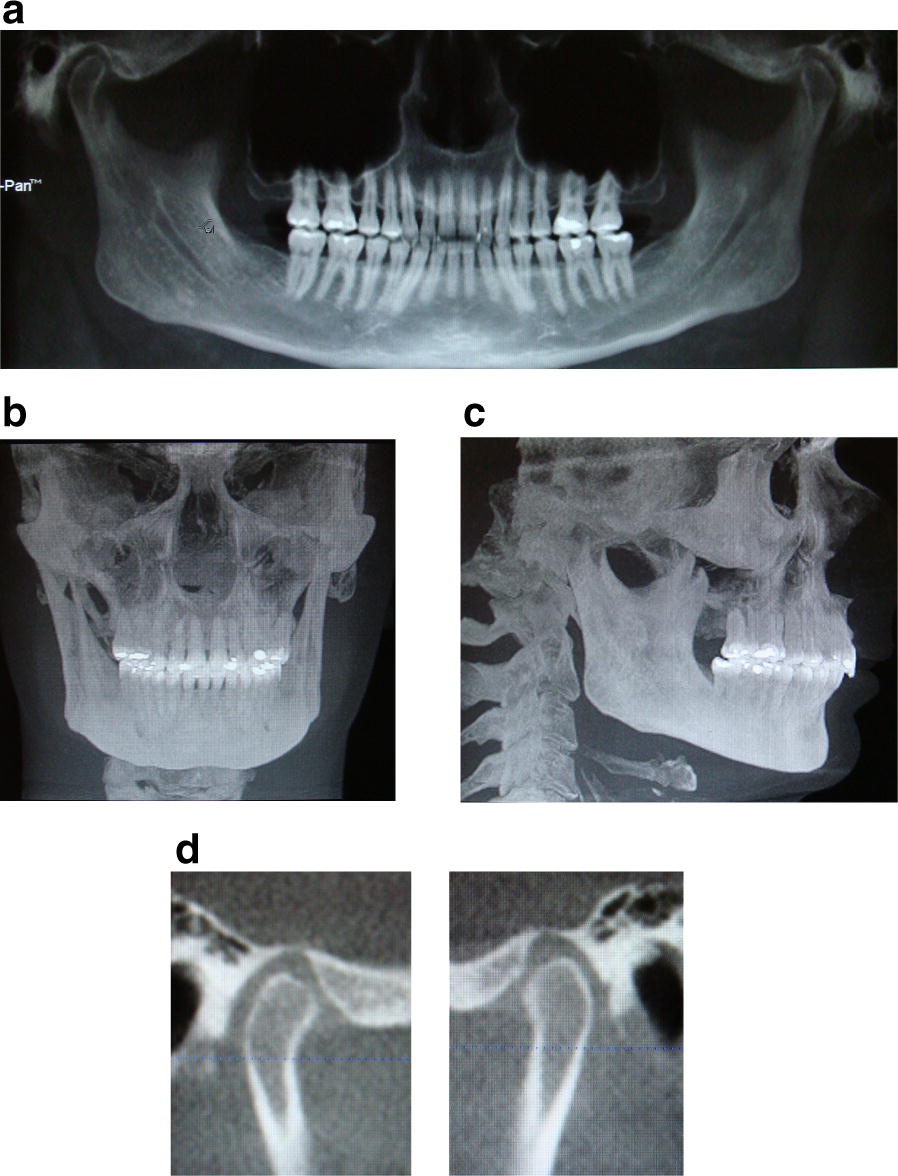
**a** Panoramic, **b** AP cephalometric, **c** lateral cephalometric, **d** right and left TMJ tomography

Diagnosis consisted of bruxism secondary to cephalgia, capsulitis and limited opening. Appliance therapy was initiated and upon his return 8 months later, his inter incisal opening had increase to 62 mm and his symptoms were greatly improved (Fig. 5).

#### fMRI study of Patient 11

**Fig. 6:**
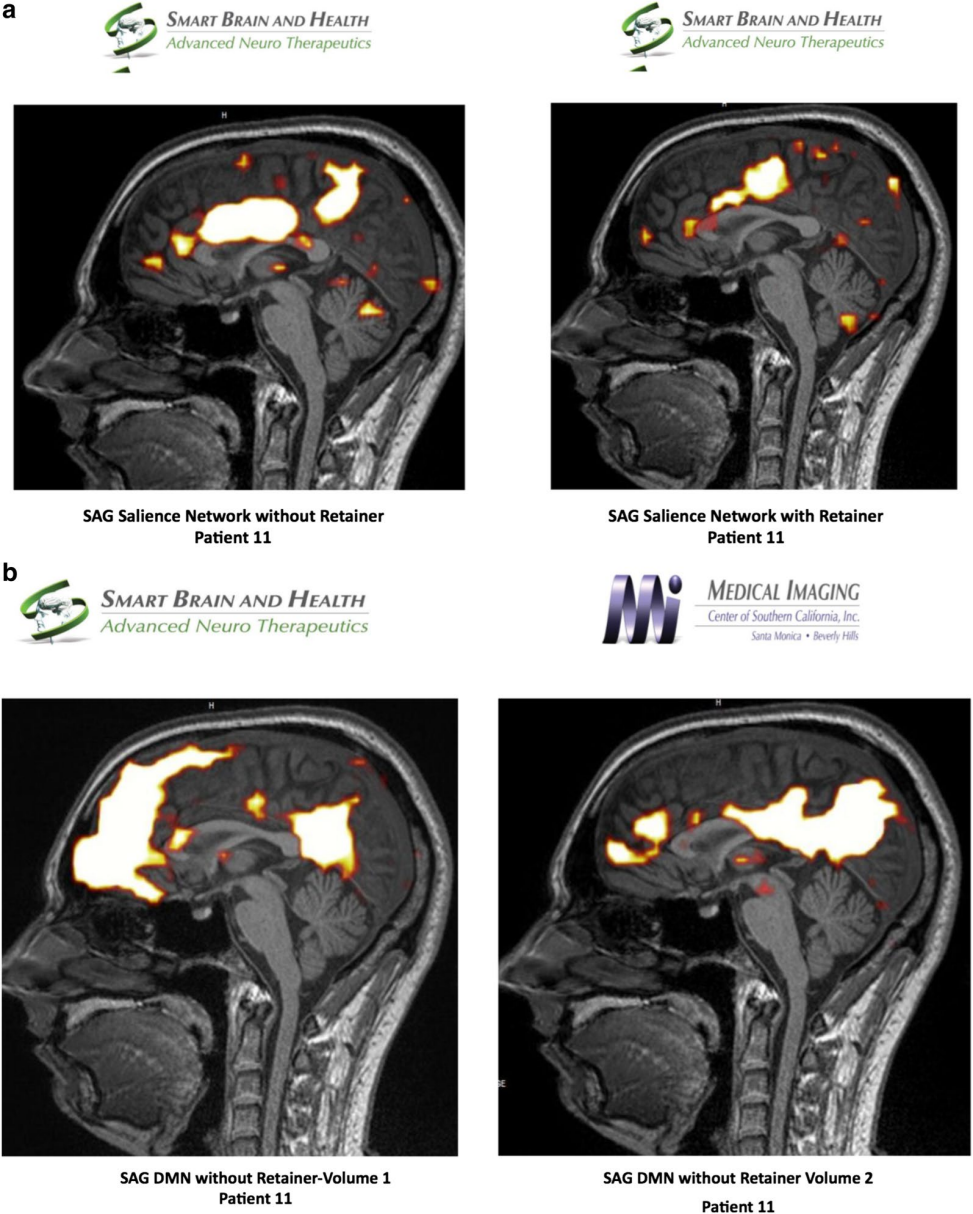

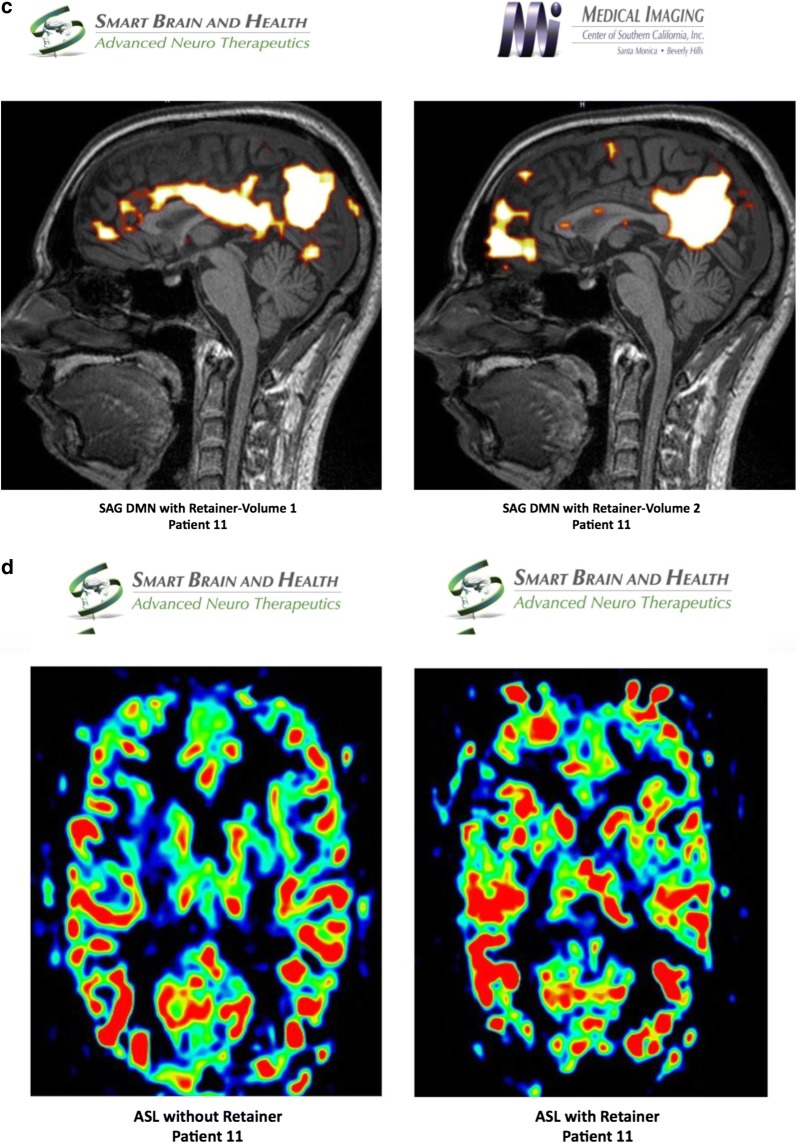
**a** Salient network analysis of patient 11 without and with dental orthotic, **b** default mode network analysis of patient 11 without dental orthotic volume 1 and 2, **c** default mode network analysis of patient 11 with dental orthotic volume 1 and 2, **d** arterial spin labeling analysis of patient 11 without and with dental orthotic

Using independent component analysis, the baseline BOLD study was evaluated and the default mode network was analyzed pre and post positioning of a patient custom-made dental orthotic. There was an abnormal default mode network with the use of the dental orthotic, inasmuch as there was a markedly hyperconnected medial orbitofrontal complex in one of the images from the default mode network. However, patient had reduced pain with dental orthotic in place (perhaps due to change in pain pathways and compensation). When compared with the default mode network without the dental orthotic, this degree of abnormality increased in the medial orbitofrontal complex in the anterior component of the default mode network. Analyzing the salience network, it revealed a marked difference between the connectivity of the salience network with the orthotic splint versus without. It is noted that once the dental orthotic was removed, the baseline BOLD signal in the salience network was markedly hyperconnected when compared with the salience network with the dental orthotic. The ASL study reveals a slightly diminished blood flow without the dental orthotic as evidenced by diminished flow to the anterior frontal lobes, left greater than right. Using diffusion weighted imaging, the FA analysis of the white matter revealed that the genu of the corpus callosum measures 0.60, the anterior body 0.25, the mid body 0.19, the posterior body 0.18, and the splenium of the corpus callosum 0.68. These findings are markedly abnormal and represent structural hypoconnectivity which may, in part, explain the markedly hyperconnected anterior component of the default mode network (Fig. 6).

## Discussion

Our hypothesis was supported by the results. The clinical diagnosis and fMRI analysis revealed relevant clinical markers and pattern of brain signature before and after orthotic use.

Patient 1: Diagnosed with capsulitis (without active bruxism at the time of examination), biomarker study showed low absorbance values. Concentration of biomarkers could not be calculated, since values were below the detection levels.

Patient 2: Elevation of IL-6 in right side synovial fluid that matches the clinical diagnosis, since active bruxism was present. The right-side joint was not functioning correctly. The condyles were translating unevenly. Upon opening, the mandible was deflected towards right for about 8 mm, which meant that it exerted considerable pressure on the right side joint, and may have resulted in compression of surrounding tissues and the disc of the joint as well as branches of auriculotemporal nerve (AT nerve).

Patient 3: Diagnosed with left TMJ disc displacement with reduction, right TMJ osteoarthrosis, crepitus and bruxism. Patient had a non-active disease where the joint was stable.

Patient 4: Full denture patient with worn down dentition. No active pathology within the joints, but had muscle spasms and pain due to the mal-occlusion from the worn-down dentition of the dentures.

Patient 5: Diagnosed with bilateral disc displacement with reduction, myalgia of the masseter and bruxism. Biomarker study showed low absorbance values for TNF-α biomarker between synovial fluid left and synovial fluid right. Concentration of biomarker could not be calculated, since values were below the detection levels. Patient had no inflammation within the joints and chronic disc displacement indicating a non-active disease process at the time of examination.

Patient 6: Elevation of IL-6 in left synovial fluid at a concentration of 70.36 pg/ml, which was significantly higher than the right side at a concentration of 0.0 pg/ml. Subject 6 also showed significant elevation of PAD-4 biomarker in both side synovial fluid at concentrations left 10,680 and right 2111.2 pg/ml. The clinical diagnosis did match the outcome of the ELISA results. The patient had bilateral degenerative joint and the left side showed a bone on bone contact (meaning active acute degeneration), which meant that this was the side that had elevated levels of IL-6, a proinflammatory cytokine, since the pressure of rubbing may still have effects on surrounding tissues. The right side was stabilized, through the process of chronic bone resorption.

Patient 7: Right synovial fluid showed significantly higher absorbance values. The clinical diagnosis showed dysfunctional joint on both sides; however, the left side showed the damage more prominently. The diagnosis matched the results (according to the kit design). The branches of AT nerve compression caused a release of SP.

Patient 8: Presented with higher absorbance values on the right side. This also matches the clinical diagnosis of the TMJ specialist, since the left condyle in patient 8 was posteriorized and there was flattening of the anterior surface of the condylar head, resulting in compression of the innervating AT nerve, thus release of SP.

Patient 9: Clinical evaluation based on a long term follow up revealed a gain in range of motion from 44 mm to 50 mm, right excursion 10 mm, left excursion 9 mm and protrusive 12 mm. Based on the VAS scale, she reported that her facial pain went from a level 9 to level 1, jaw pain reduced from 9 to 1, neck pain reduced from 10 to 1, headaches reduced from 8 to 0, migraine reduced from 10 to 0, visual disturbances reduced from 6 to 3, dystonia reduced from 6 to 0, eye pain and pain to chewing had resolved, dizziness improved 98% and ringing in the ears reduce by 80%.

Functional MRI revealed changes in metabolism and/or blood flow in the bifrontal areas with diminished flow when scanned without dental orthotic. There was no significant change in the default mode network pre-and post-positioning of dental orthotic. Marked discrepancy was observed in the salience network, as noted in the description between with and without dental orthotic study.

Patient 10: Clinical evaluation based on a long term follow up revealed an improvement in range of motion from 40 mm to 50 mm, right excursion 9 mm, left excursion 8 mm and protrusive 10 mm. The patient reported an improvement in symptoms: Migraines, muscle twitching and visual disturbances had resolved; shoulder pain improved by 70%; neck pain improved 60%; back pain, dizziness and headaches improved 45%; fatigue improve 30%, throat pain had no change.

Increased connectivity was observed in the salience network study without a dental orthotic in a patient who was experiencing considerable pain. The salience network would return to a normal footprint when the dental orthotic was used. ASL study revealed diminished flow and hypometabolism in the anterior frontal lobe that is most noticeable on the studies without the retainer suggesting that when the patient is in pain, there is diminished flow to the frontal lobe.

Patient 11: Clinical evaluation based on a long term follow up revealed an improvement in range of motion from 40 mm to 65 mm, right excursion 10 mm, left excursion 12 mm and protrusive 11 mm. The patient reported improvement in symptoms during a follow up visit before making adjustments to the orthotic: Ear pain resolved; eye pain improved 90%; headaches and sinus congestion improved 80%; ear congestion, jaw pain and pain when chewing improved 75%; facial pain and back pain improved 70%; jaw joint pain, pain at hands and feet, migraine and throat pain improved 50%; fatigue improved 40%; neck and shoulder pain were the same. After an adjustment to the orthotic during the visit, reported improvement of symptoms to 0–1 based on the VAS scale.

During the fMRI process, the patient reported of facial pain, neck pain and upper back pain when the orthotic was not in his mouth and resolution of the pains during the second fMRI when the orthotic was place in his mouth. There was an increase in connectivity in the medial orbitofrontal component of the anterior default mode network without the dental orthotic. The salience network analysis revealed that there was markedly increased connectivity in the salience network once the dental orthotic was removed, which correlates with the patient’s symptoms of pain and discomfort returning once the orthotic was removed. Further analysis would be required to clarify whether this hyperconnected salience network is a marker for returning pain. ASL study revealed decreased blood flow [and possibly decreased metabolism] once the dental orthotic was removed. There are markedly abnormal FA measurements of the white matter tract as evidenced by measurements of the body of corpus callosum, which may represent long term structural hypoconnectivity abnormalities in this patient’s brain.

## Conclusion

This study has revealed that TMD patients with neurological comorbidities show common patterns of signature changes in brain function. The alterations in the thalamocortical pathway differed between the fMRI studies of before and after oral orthotic insertion. Our findings highlight the potential for brain neuroimaging as an investigative tool for understanding pathological changes in the TMJ positioning and development of TMD, which can serve as a source of peripheral neuropathy causing inflammatory changes in the CNS. The fMRI proves that as we remove the source of peripheral inflammation, we see instant significant changes in brain activity and signal overload.

## Data Availability

Data sharing is not applicable to this article as no datasets were generated or analysed during the current study.

## Abbreviations

TMJ: temporomandibular joint
TMD: temporomandibular joint disorders
PNS: peripheral nervous system
CNS: central nervous system
fMRI: functional magnetic resonance imaging
AT: auriculotemporal
CRPS: complex regional pain syndrome
RSD: reflex sympathetic dystrophy
TG: trigeminal ganglion
SGCs: satellite glial cells
BOLD: blood oxygenation level dependent
ICA: independent components analysis
ICNs: intrinsic connectivity networks
DMN: default mode network
ASL: arterial spin labeling
CBV: cerebral blood volume
CBF: cerebral blood flow
MTT: mean transit time
DTI: diffusion tensor imaging
ADC: apparent diffusion coefficient
FA: fractional anisotropy
JVA: joint vibration analysis
EMG: electromyography
CBCT: cone beam computerized tomography
VAS: visual analog scale
MPRAGE: magnetization prepared rapid gradient-echo
